# Improvement of long-term outcomes in pancreatic cancer and its associated factors within the gemcitabine era: a collaborative retrospective multicenter clinical review of 1,082 patients

**DOI:** 10.1186/1471-230X-13-134

**Published:** 2013-08-31

**Authors:** Taira Kuroda, Teru Kumagi, Tomoyuki Yokota, Hirotaka Seike, Mari Nishiyama, Yusuke Imai, Nobu Inada, Naozumi Shibata, Satoshi Imamine, Shin-ichi Okada, Mitsuhito Koizumi, Hirofumi Yamanishi, Nobuaki Azemoto, Jiro Miyaike, Yoshinori Tanaka, Haruka Tatsukawa, Hiroki Utsunomiya, Yoshinori Ohno, Teruki Miyake, Masashi Hirooka, Shinya Furukawa, Masanori Abe, Yoshiou Ikeda, Bunzo Matsuura, Yoichi Hiasa, Morikazu Onji

**Affiliations:** 1 Department of Gastroenterology and Metabology, Ehime University Graduate School of Medicine, Shitsukawa, To-on, Ehime 791-0295, Japan; 2 Internal Medicine, Saiseikai Imabari Hospital, Imabari, Ehime 799-1592, Japan; 3 Center for Liver-Biliary-Pancreatic Disease, Matsuyama Red Cross Hospital, Matsuyama, Ehime 790-8524, Japan; 4 Gastroenterology, Uwajima Municipal Hospital, Uwajima, Ehime 798-8510, Japan; 5 Gastroenterology, Matsuyama Municipal Hospital, Matsuyama, Ehime 790-0067, Japan; 6 Gastroenterology, Ehime Prefectural Central Hospital, Matsuyama, Ehime 790-0024, Japan; 7 Internal Medicine, Saiseikai Matsuyama Hospital, Matsuyama, Ehime 791-8026, Japan; 8 Gastroenterology, Ehime Prefectural Niihama Hospital, Niihama, Ehime 792-0042, Japan; 9 Internal Medicine, Ohzu Municipal Hospital, Ohzu, Ehime 795-0013, Japan; 10 Internal Medicine, Saiseikai Saijo Hospital, Saijo, Ehime 793-0027, Japan; 11 Endoscopy Center, Ehime University Hospital, Ehime, Ehime 791-0295, Japan

**Keywords:** Pancreatic cancer, Chemotherapy, Best supportive care, Gemcitabine, Long-term outcome, Japan

## Abstract

**Background:**

Although the outcomes of pancreatic cancer have been improved by gemcitabine, the changes in its characteristics and long-term outcomes within the gemcitabine era remain unclear. This study was conducted to identify clinical characteristics of pancreatic cancer patients within the gemcitabine era.

**Methods:**

A retrospective chart review was performed at 10 centers for 1,248 consecutive patients who were ever considered to have a diagnosis of pancreatic cancer between 2001 and 2010. Data collected included demographics, diagnosis date, clinical stage, treatment, and outcome; 1,082 patients met the inclusion criteria and were analyzed further. The chi-square test, Student’s t-test, and Mann–Whitney U-test were used for statistical analysis. Outcomes were analyzed using the Kaplan-Meier method and Cox proportional hazards regression. Differences in survival analyses were determined using the log-rank test.

**Results:**

The distribution of clinical stages was: I, 2.2%; II, 3.4%; III, 13%; IVa, 27%; and IVb, 55%. Chemotherapy alone was administered to 42% of patients and 17% underwent resection. The 1-, 3-, and 5-year survival rates were 39%, 13%, and 6.9%, respectively. The median survival time was 257 days, but differed considerably among treatments and clinical stages. Demographics, distribution of clinical stage, and cause of death did not differ between groups A (2001–2005, n = 406) and B (2006–2010, n = 676). However, group B included more patients who underwent chemotherapy (P < 0.0001) and fewer treated with best supportive care (P = 0.0004), mirroring improvements in this group’s long-term outcomes (P = 0.0063). Finally, factors associated with long-term outcomes derived from multivariate analysis were clinical stage (P < 0.0001), location of the tumor (P = 0.0294) and treatments (surgery, chemotherapy) (P < 0.0001).

**Conclusions:**

Long-term outcomes in pancreatic cancer has improved even within the gemcitabine era, suggesting the importance of offering chemotherapy to patients previously only considered for best supportive care. Most patients are still diagnosed at an advanced stage, making clinical strategy development for diagnosing pancreatic cancer at earlier stages essential.

## Background

Pancreatic cancer (PC) has one of the worst prognoses among cancers worldwide (the overall 5-year survival rate <5%), and the number of patients with PC is increasing globally [1]. For example, PC is currently the fifth leading cause of cancer death in Japan for each sex. The management of PC has changed since gemicitabine (GEM) was introduced worldwide in 1996. Nevertheless, the prognosis for PC remains extremely poor due to a lack of effective strategies for early detection of the disease [2]. In 2007, the Japanese Pancreas Society (JPS) conducted a nationwide survey analyzing over 24,000 patients with PC between 1981 and 2004. The results showed an improvement in long-term outcomes over the course of 3 different eras (the 1980s, 1990s, and the GEM era [which for Japan began in 2001]). Improvements in outcome were largely attributed to an increase in the resection rate (1980s vs. 90s) and the emergence of GEM (1990s vs. GEM era) [3]. In addition, using their nationwide PC registry, the JPS has extended the analysis to include patients through 2007 (>32,000 cumulative records) and has confirmed that survival improvements in PC can be attributed to chemotherapy (primarily GEM) [4]. However, the changes in the characteristics and outcomes of this disease in Japan within the GEM era alone have not been definitively established. Furthermore, guideline compliance of PC in routine clinical practice is unknown, especially whether GEM is administered to unresectable PC as first-line therapy.

In this context, the Ehime Pancreato-Cholangiology (EPOCH) Study Group was established. This group conducted a retrospective study to identify the clinical characteristics of PC in Japan within the GEM era (after 2001).

## Methods

A retrospective chart review was conducted and included 1,248 consecutive patients who were ever considered to have a diagnosis of PC at the gastroenterology clinic at Ehime University Hospital or one of its 9 affiliated hospitals (EPOCH Study Group), between January 2001 and December 2010. As of August 2011, data collected included demographics (age and sex), date of diagnosis, tumor location, clinical stage (JPS TNM classification [5]), treatment, and outcome. Briefly, it must be noted the difference of TNM classification between JPS and UICC is in the staging system based on the definition of tumor factors (T) and lymph node metastases (N). T4 in UICC indicates tumor invasion limited to the trunk of the celiac artery or superior mesenteric artery, whereas invasion to any major vessels, neural plexus, and adjacent organs are included in the JPS classification. Positive lymph node metastasis in UICC is N1, while in JPS there exist various grades depending on the distance from the main tumor (N1, N2, N3). PC was diagnosed on the basis of abdominal imaging (computed tomography, conventional ultrasonography, and magnetic resonance imaging) reported by radiologists with or without histologic findings (needle biopsy specimens obtained for suspicion of liver metastasis obtained under ultrasonography, fine needle aspiration biopsy specimens obtained under endoscopic ultrasonography, or surgical specimens) reported by pathologists. After an extensive chart review, the final diagnosis with intraductal papillary mucinous neoplasm (n = 22), neuroendocrine tumor (n = 5), small cell carcinoma (n = 2), serous adenocarcinoma (n = 1), and undifferentiated carcinoma (n = 1) were excluded from the analysis. Patients with missing data (n = 135) were also excluded from the analysis. For patients who did not undergo surgical resection, the final clinical stage was determined on imaging. Otherwise, for patients who underwent surgical resection of the pancreas, the final clinical stage was determined at the time of pathologic analysis of the surgically resected specimen. The treatments were divided into 3 categories: surgical resection (with or without adjuvant chemotherapy), chemotherapy, and best supportive care (BSC). In total, data from 1,082 patients (87%) were analyzed. For the purpose of this study, the patients were divided into 2 groups: Group A (2001–2005, n = 406) and Group B (2006–2010, n = 676). Since only a small number of patients received radiation therapy and its proportion did not differ between Group A (n = 36, 8.9%) and Group B (n = 45, 6.7%) (P = 0.1810), these patients were included in the treatment groups corresponding to their main treatment: surgical resection (n = 10), chemotherapy (n = 63), and BSC (n = 8).

The chi-square test, Student’s t-test, and Mann–Whitney U-test were used for statistical analysis, where appropriate. Outcomes were analyzed using the Kaplan-Meier method and Cox proportional hazards regression. Differences in survival analyses were determined using the log-rank test. Two-tailed significance was defined in all analyses as a P-value < 0.05. All statistical analyses were performed using a statistical software package (JMP, version 8; SAS Institute, Cary, NC). Data were stored in a secure database and patients were numerically coded to anonymize data. The study protocol conformed to the ethical guidelines of the 1975 Declaration of Helsinki and was approved by the local ethics committee at the Ehime University Graduate School of Medicine.

## Results

### Demographic features of the entire cohort

**Figure 1:**
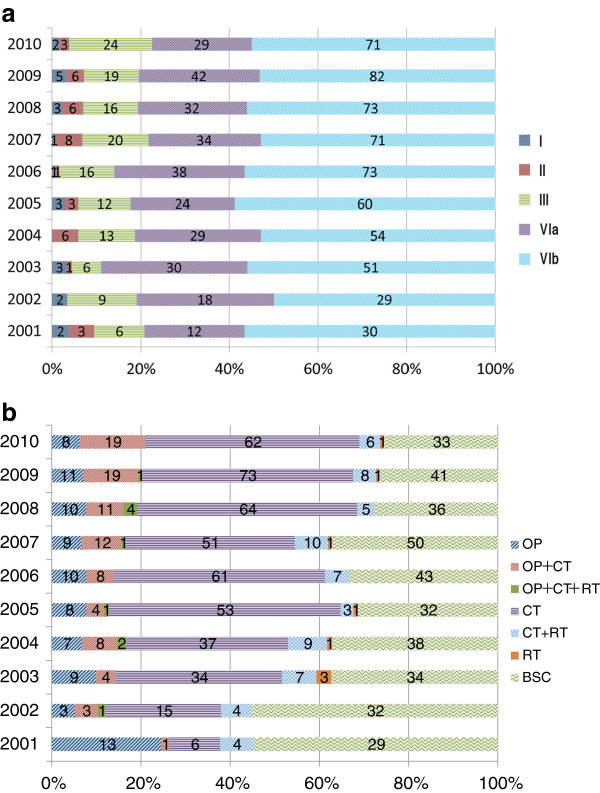
**Changes in the clinical stages and treatments in pancreatic cancer. a**. Changes in the clinical stages of patients with pancreatic cancer between 2001 and 2010 (n = 1,082). **b**. Changes in treatments for patients with pancreatic cancer between 2001 and 2010 (n = 1,082). OP, operation; CT, chemotherapy; RT, radiation therapy; BSC, best supportive care.

The mean ± standard deviation for the age of male subjects included in the cohort was 69 ± 11 years (range, 33–91 years, n = 566) and 74 ± 11 years (range, 37–86 years, n = 516) for female subjects. The female subjects were significantly older than the male subjects (P < 0.0001). The distribution of clinical stages was as follows: Stage I (n = 22, 2.2%), II (n = 37, 3.4%), III (n = 141, 13%), IVa (n = 288, 27%), and IVb (n = 594, 55%). This distribution did not change significantly over time: Stage I, 0–3.8%; Stage II, 0–6%; Stage III, 6.6–19%; Stage IVa, 23–33%; and Stage IVb, 50–59% (Figure 1a). The mean age of patients did not differ among clinical stages: 75 ± 13 years, 73 ± 11 years, 71 ± 11 years, 72 ± 11 years, and 71 ± 11 years for Stages I, II, III, IVa, and IVb, respectively. Tumors were located mostly in the head of the pancreas (n = 634, 59%), followed by the body (n = 235, 22%) and the tail (n = 130, 12%) of the pancreas. The association between clinical stage and tumor location was analyzed in 999 patients (92.3%) after 83 patients who had tumors that were either difficult to locate (n = 34, 3.1%) or that had expanded to multiple sites (n = 49, 4.5%) were excluded. Stage IVb included significantly fewer patients with tumors in the head of the pancreas (P < 0.0001), whereas more patients with Stage IVb cancer had tumors in the tail of the pancreas than patients who did not have Stage IVb disease: Stage IVb (n = 529; tumor location: head 54%, n = 284; tumor location: body 26%, n = 137; tumor location: tail 20%, n = 108) vs. non-Stage IVb disease (n = 470; tumor location: head 75%, n = 350; tumor location: body 21%, n = 98; tumor location: tail 4.7%, n = 22).

### Treatments for the entire cohort

From the entire cohort, 42% (9%, 8%, 16%, 35%, and 46% of the patients in Stages I, II, III, IVa, and IVb, respectively) underwent chemotherapy alone (GEM-based, 88%) and the number of patients receiving chemotherapy increased over time (Figure 1b). GEM was chosen as a first-line regimen in 81% of the patients, followed by S-1 (12%) and 5-fluorouracil (1%). Only 187 patients (17%) were able to undergo surgical resection (32%, 51%, 60%, 19%, and 3.5% of the patients in Stages I, II, III, IVa, and IVb, respectively), and adjuvant chemotherapy was given to 53% (n = 100) of these patients. Notably, 11 patients (50%) and 11 patients (30%) in Stages I and II, respectively, chose BSC. The primary reasons for this choice were: patient and family preference (n = 6), advanced age (n = 3), poor performance status (n = 3), serious complications (n = 2), and unknown (n = 8).

### Long-term outcomes for the entire cohort

**Figure 2:**
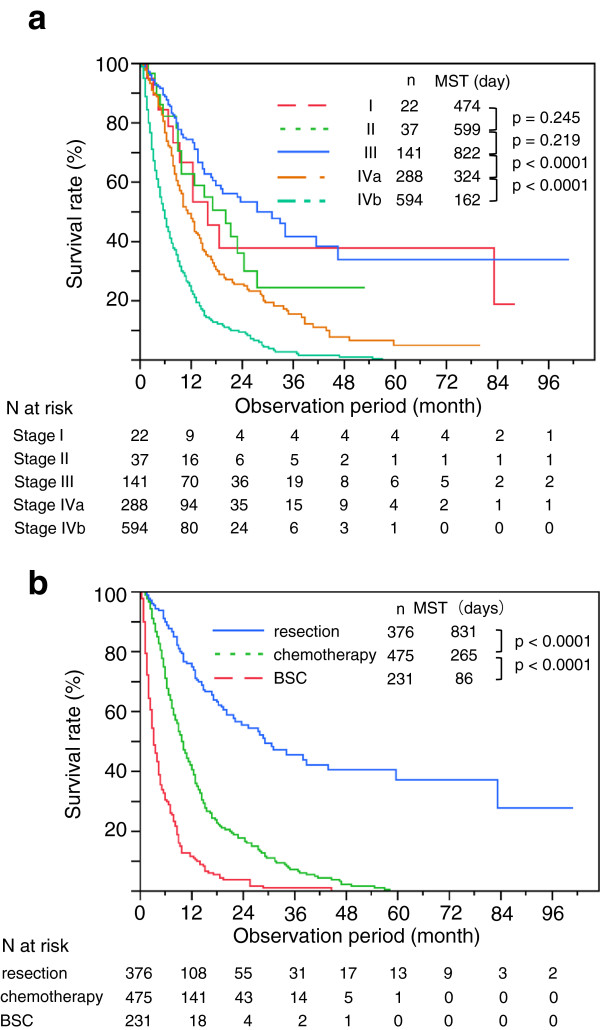
**Comparison of survival curves in pancreatic cancer. a**. Comparison of survival curves among clinical stages in patients with pancreatic cancer (n = 1,082). MST, median survival time. **b**. Comparison of survival curves among different treatments in patients with pancreatic cancer (n = 1,082). MST, median survival time; OP, operation; CT, chemotherapy; BSC, best supportive care.

During the mean observation period of 288 ± 374 days (range, 1–3052 days; median, 176 days), 685 patients died. The cause of death was identified in 629 patients (92%) and was related to PC in 95.1% (n = 598). Only 4.9% (n = 31) of deaths were unrelated to PC (i.e., pneumonia, n = 4; cardiac disease, n = 4; upper gastrointestinal hemorrhage, n = 2; other, n = 21). Overall, the 1-, 3-, and 5-year survival rates were 39%, 13%, and 6.9%, respectively. The median survival time (MST) of the entire cohort was 257 days but differed significantly among clinical stages (474 days, 599 days, 822 days, 324 days, and 162 days for Stages I, II, III, IVa, and IVb, respectively; P < 0.0001 between Stages III and IVa and between Stages IVa and IVb; Figure 2a). Significant differences were also found in MST with respect to tumor location (MST for tumors involving the head of the pancreas, 287 days; MST for tumors not involving the head, 230 days; P < 0.0001) and with respect to treatments (831 days, 265 days, and 86 days for resection, chemotherapy, and BSC, respectively; P < 0.0001; Figure 2b).

Analysis comparing the 2 groups (Group A and Group B) did not show any significant differences in demographics (age and sex), distribution of clinical stage, and cause of death (PC-related vs. PC-unrelated). Although the resection rate did not differ between Group A and Group B (16% vs. 18%; P = 0.306), Group B (n = 148) included more patients with Stage III disease (46% vs. 36% in group A) and fewer with Stage IVa disease (28% vs. 45% in Group A; P = 0.012). Group B also included more patients treated with chemotherapy and fewer with BSC than Group A (chemotherapy, 51% vs. 42%; P < 0.0001, BSC, 31% vs. 42%; P = 0.0004) (Table 1).

**Table 1:** Comparison of the baseline demographics between 2001–2005 and 2006–2010 in patients with pancreatic cancer (n = 1,082)

		**Total**	**2001–2005**	**2006–2010**	**P-value**
Age (y)		71.5 ± 10.9	71.0 ± 10.4	71.8 ± 11.2	0.2491^1^
Sex (Male/Female)		566/516	210/196	356/320	0.7647^2^
Stage	I	22 (2.0%)	10 (2.5%)	12 (1.8%)	0.4376^2^
	II	37 (3.4%)	13 (3.2%)	24 (3.6%)	0.7602^2^
	III	141 (13.0%)	46 (11.3%)	95 (14.0%)	0.1976^2^
	IVa	288 (26.6%)	113 (27.8%)	175 (25.9%)	0.4834^2^
	IVb	594 (54.9%)	224 (55.2%)	370 (54.7%)	0.8883^2^
Treatment	Surgery	187 (17.3%)	64 (15.8%)	123 (18.2%)	0.3057^2^
	Chemotherapy	519 (48.0%)	172 (42.4%)	347 (51.3%)	<0.0001^2^
	BSC/RT	376 (34.8%)	170 (41.9%)	206 (30.5%)	0.0004^2^
Cause of death		654/31	274/12	380/19	0.7252^2^
PC related/PC unrelated					

BSC, best supportive care; RT, radiation therapy; PC, pancreatic cancer.

^1^Student’s t-test; ^2^chi square test.

**Figure 3:**
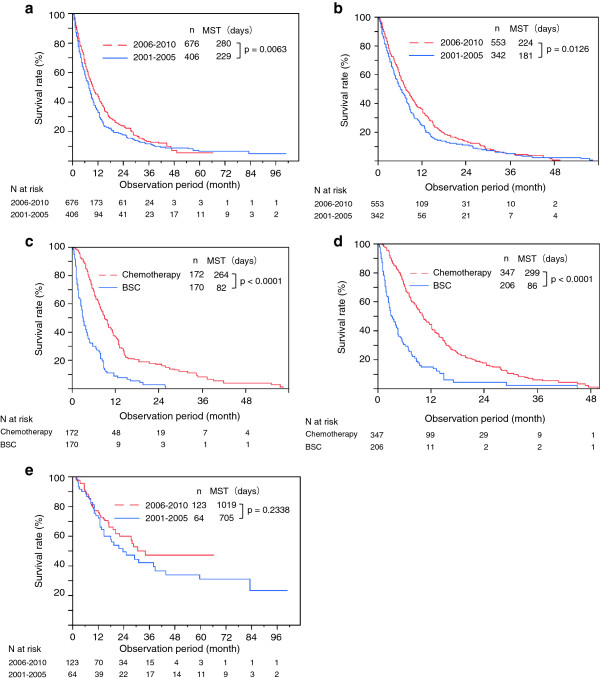
**Comparison of survival curves in pancreatic cancer. a**. Comparison of survival curves between Group A (2001–2005) and Group B (2006–2010) in patients with pancreatic cancer (n = 1,082). MST, median survival time. **b**. Comparison of survival curves between Group A (2001–2005) and Group B (2006–2010) in patients with pancreatic cancer who chose chemotherapy or best supportive care (n = 895). MST, median survival time. **c**. Comparison of survival curves between patients with pancreatic cancer who chose chemotherapy and best supportive care in Group A (2001–2005) (n = 342). MST, median survival time. **d**. Comparison of survival curves between patients with pancreatic cancer who chose chemotherapy and best supportive care in Group B (2006–2010) (n = 553). MST, median survival time. **e**. Comparison of survival curves between Group A (2001–2005) and Group B (2006–2010) in patients who had pancreatic cancer and underwent surgical resection (n = 187). MST, median survival time.

The long-term outcome of Group B was significantly better than that of Group A (MST: Group A, 229 days vs. Group B, 280 days; P = 0.0063; Figure 3a). The entire unresected group, patients who chose chemotherapy or BSC, significantly improved its long-term outcome (N = 895) (MST: Group A, 181 days vs. Group B, 224 days; P = 0.0063; Figure 3b). Sub-analyses also showed a significant improvement in the 2 groups when chemotherapy was given (MST in Group A: chemotherapy, 264 days vs. BSC, 82 days; P < 0.0001; Figure 3c) (MST in Group B: chemotherapy, 299 days vs. BSC, 86 days; P < 0.0001; Figure 3d). However, long-term outcomes did not differ statistically between the 2 groups when compared separately with respect to chemotherapy (MST: Group A, 264 days vs. Group B, 299 days; P = 0.5476) and BSC (MST: Group A, 82 days vs. Group B, 86 days; P = 0.3949). On the other hand, the surgically resected group did not improve its long-term outcome (N = 187) (MST: Group A, 705 days vs. Group B, 1019 days; P = 0.2338; Figure 3e). Although Group B also contained more patients who received adjuvant chemotherapy compared to Group A (61% vs. 38%: P = 0.0023), long-term outcomes for Group A (MST: with adjuvant chemotherapy, 749 days vs. without adjuvant chemotherapy, 532 days; P = 0.5037), and Group B (MST: with adjuvant chemotherapy, 438 days vs. without adjuvant chemotherapy, 273 days; P = 0.2271) did not differ between patients who received adjuvant chemotherapy and those who did not. Additional analysis evaluating a benefit of adjuvant chemotherapy among different clinical stage groups (stage I - III, stage IVa) failed to show its benefit over surgery alone: stage I - III (P = 0.1335) and stage IVa (P = 0.2424).

### Factors associated with long-term outcomes

Finally, the factors associated with long-term outcomes in the 1,082 patients with PC were analyzed. According to univariate analysis, variables significantly associated with better survival were younger age (HR 0.80; 95% CI, 0.67 – 0.94; P = 0.0072), earlier stages (stage I – III vs. stage IVa - IVb: HR 0.32; 95% CI, 0.25 – 0.40; P < 0.0001), tumor location (head vs. non-head: HR 0.77; 95% CI, 0.66 – 0.91; P = 0.0015), treatments [(surgery vs. chemotherapy: HR 0.31; 95% CI, 0.24 – 0.40; P < 0.0001), (surgery vs. BSC: HR 0.11; 95% CI, 0.08 – 0.14; P < 0.0001), (chemotherapy vs. BSC: HR 0.34; 95% CI, 0.29 – 0.40; P < 0.0001)], and the period diagnosed as PC (Group B vs. Group A: HR 0.81; 95% CI, 0.69 – 0.94; P = 0.0063) (Table 2). Furthermore, multivariate analysis showed that earlier stages (stage I – III vs. stage IVa - IVb: HR 0.46; 95% CI, 0.36 – 0.59; P < 0.0001), tumor location (head vs. non-head: HR 0.83; 95% CI, 0.71 – 0.98; P = 0.0294), and treatments [(surgery vs. chemotherapy: HR 0.43; 95% CI, 0.33 – 0.56; P < 0.0001), (surgery vs. BSC: HR 0.13; 95% CI, 0.10 – 0.18; P < 0.0001), (chemotherapy vs. BSC: HR 0.31 95% CI, 0.26 – 0.37; P < 0.0001)] were significantly associated with long-term outcomes (Table 2). However, age (P = 0.4581) and the period diagnosed as PC (P = 0.2357) did not associate with long-term outcomes.

**Table 2:** Factors associated with long-term outcomes pancreatic cancer derived from univariate analysis and multivariate analysis (n = 1,082)

	**Hazard ratio**	**95% CI**	**P-value**
Univariate analysis			
Age < 65 years old	0.80	0.67 ‒ 0.94	0.0072
Female	0.95	0.82 ‒ 1.11	0.5648
Surgery (vs. Chemotherapy)	0.31	0.24 ‒ 0.40	<0.0001
Surgery (vs. BSC)	0.11	0.08 ‒ 0.14	<0.0001
Chemotherapy (vs. BSC)	0.34	0.29 ‒ 0.40	<0.0001
Tumor Location (Head)	0.77	0.66 ‒ 0.91	0.0015
Group B (2006–2010)	0.81	0.69 ‒ 0.94	0.0063
Stage I‒III	0.32	0.25 ‒ 0.40	<0.0001
Multivariate analysis			
Age < 65 years old	0.94	0.79 ‒ 1.11	0.4581
Surgery (vs. Chemotherapy)	0.43	0.33 ‒ 0.56	<0.0001
Surgery (vs. BSC)	0.13	0.10 ‒ 0.18	<0.0001
Chemotherapy (vs. BSC)	0.31	0.26 ‒ 0.37	<0.0001
Tumor Location (Head)	0.83	0.71 ‒ 0.98	0.0294
Group B (2006–2010)	0.91	0.78 ‒ 1.06	0.2357
Stage I‒III	0.46	0.36 ‒ 0.59	<0.0001

BSC, best supportive care.

## Discussion

This multicenter retrospective study performed in gastroenterology clinics (EPOCH Study Group) in Japan, reports findings for 1,082 patients with PC. The results of this study support previously reported findings in that (1) a vast majority of patients with PC are still diagnosed at advanced stages; (2) patient prognosis in PC remains extremely poor; (3) tumors located in the pancreatic head are encountered most often at diagnosis; (4) tumors located in the tail are more often diagnosed at Stage IVb; (5) patients with PC involving the pancreatic head have a better prognosis than those with tumors in the body or tail; and (6) very few patients with PC may expect long-term survival, unless diagnosed at an earlier stage and treated with surgical resection. More importantly, this study was the first to observe and identify changes in the clinical characteristics of PC in a large number of patients within the GEM era.

Overall, this study showed an improvement in long-term outcomes within the GEM era, and the associated factors for were clinical stage, tumor location and treatments (surgery and chemotherapy). The former finding is likely to be related to more patients being given an opportunity to undergo chemotherapy (the majority with a GEM-based regimen as first-line treatment) and fewer patients choosing BSC in the second half of the study period. Although not investigated, this was presumably due to physician’s skill up in chemotherapy rather than the change of patient characteristics, and indeed the referral pattern did not change during the study period. The improvement in MST differed by only 51 days between Group A (2001–2005) and Group B (2006–2010). However, considering that MST in Group A was as short as 229 days and PC is associated with one of the worst prognoses among cancers worldwide, it is important to appreciate the role of chemotherapy in the treatment of PC. Significant improvement in long-term outcomes was not seen over time (Group A vs. Group B) for chemotherapy (MST, 264 vs. 299 days). However, patients who were given chemotherapy had significantly better long-term outcomes than those who chose BSC in the 2 groups. Significant improvement in long-term outcomes was also not seen over time for surgical resection (MST, 705 vs. 1019 days). This may be due to a small number of patients for surgical resection in our study population among gastroenterology clinics. Notably, more than 20% of patients with PC still chose BSC. In general, quality of life (QOL) is one of the most important factors when patients are considering chemotherapy as a treatment option, but this study did not investigate QOL data. Therefore, this study cannot evaluate changes in QOL with either chemotherapy or BSC. Nevertheless, a linear increase in the number of patients choosing chemotherapy and a concomitant decrease in patients choosing BSC were seen (Figure 1b).

The cohort included in this study was unique because 11 patients (50%) in Stage I and 11 patients (30%) in Stage II chose BSC, which led to a poorer prognosis associated with these stages. This observation was likely due to referral patterns in this retrospective study conducted among gastroenterology clinics (i.e., these patients were not referred to surgeons but directly to gastroenterologists). The results of this study could not help identify the precise reasons in all patients in Stages I and II who did not undergo resection or chemotherapy. Previous studies have clearly shown that surgical resection improves prognosis among patients with locally advanced PC [6,7]. However, only a few studies have investigated the benefits of surgery among elderly patients and none of these studies were large. Therefore, the ability to extrapolate these data to an elderly population remains controversial [8]. It is also important to identify the role of chemotherapy in the elderly. Yamagishi, et al. showed that elderly patients (>75 years of age) who had good performance status and were treated with GEM had significantly better long-term outcomes without serious adverse events than those who did not receive any treatment [9]. However, like the current study, this study was retrospective. A prospective study is necessary to identify the true impact of chemotherapy in elderly patients with PC. This suggests that in order to further improve long-term outcomes in PC, it is important to offer treatment to patients who were previously only considered for BSC. Some reports have claimed that other types of cancer are undertreated in elderly patients because of clinician preference and patient bias [10-12]. Therefore, it is important to provide accurate information about treatments for PC, especially to elderly patients.

The strengths of this study include a large number of consecutive patients, the inclusion of gastroenterology clinics located in teaching, academic, and community hospitals, and an observation period limited to the GEM era. All of these strengths support generalization of these findings as they represent the current state of routine healthcare service. Also our patients were much older than the previous national study (age; 64 vs. 69 in men, 66 vs. 74 in women) [3]. This may foresee our future clinical practice in entire Japan where aging is becoming an issue. In contrast, as with all retrospective studies, the results of this study must be viewed in light of the limitations. Since the principle aim of this study was to identify the broad picture of changes in the clinical characteristics of PC within the GEM era, other detailed data (e.g., performance status, family history, medical history, associated diseases, presentation pattern, and laboratory tests including biochemical, tumor markers, and histology) were not collected. Most importantly, we assumed that not all patients had histologically-proven adenocarcinoma, which may change the treatment strategy and prognosis. Additionally, even with a small number of patients, we might have included non-malignant cases. However, these possible cases are usually a candidate for surgical resection and given a final histological diagnosis. Indeed, histologic examination was studied in only 54.5% of patients, even in a nationwide analysis conducted by JPS [3]. Nevertheless, given that the vast majority (87%) of those Japanese patients with PC who underwent histologic examination were diagnosed with adenocarcinoma [3], this may have only a small impact on treatment strategy and prognosis in the current study population as well. In this regard, fine needle aspiration biopsy in the future may play a cardinal role for patients (those undergoing chemotherapy in particular), despite the fact that this procedure was performed at very few institutes until its nationwide approval in Japan in 2011. Additionally, one may argue that data related to the details of the chemotherapy regimens were not included (e.g., treatment response, adverse effects, and QOL during treatment). Therefore, it will be important to obtain these data when future prospective studies are conducted to further investigate clinical features that may change the management and eventually the prognosis of PC.

Although a significant improvement in the long-term outcomes of PC was achieved over the decade included in this study, it is important to stress that the clinical stage at diagnosis did not change over the same period (Figure 1a). As reported previously [3], Stages I and II represented only 5.6% of the patients presenting throughout the observation period. An effective clinical strategy for the diagnosis of PC at earlier stages is urgently required and several screening programs have been attempted, especially among individuals with a family history of PC [10,11]. However, to date, no program has been shown to effectively identify patients with PC at earlier stages [13,14].

## Conclusions

This study showed that long-term outcomes in pancreatic cancer have improved even within the GEM era. These results suggest the importance of offering chemotherapy to patients previously only considered for BSC. Nevertheless, the vast majority of patients are still diagnosed at an advanced stage, making an effective clinical strategy for the diagnosis of PC at earlier stages a crucial requirement.

## Abbreviations

PC: Pancreatic cancer; GEM: Gemcitabine; JPS: Japan pancreas society; BSC: Best supportive care; MST: Median survival time; QOL: Quality of life.

## Competing interests

The authors declare that they have no competing interests.

## Authors’ contributions

**Guarantor of the article**: Teru Kumagi, MD, PhD. **Specific author contributions**: Conception and design of the study, generation, assembly, analysis of data, interpretation of data and statistical analysis: TKur, TKum, MK, HY, NA, TM MH; data collection from each center: TKur, TKum, TY, HS, MN, YI, NI, NS, SI, SO, HT, HU, YT, JM, YO; and drafting of the paper: TKur, TKum, YH, MO. Approval of the final draft submitted was undertaken by all authors. All authors read and approved the final manuscript.

## Pre-publication history

The pre-publication history for this paper can be accessed here:

http://www.biomedcentral.com/1471-230X/13/134/prepub
